# HIV Testing and PrEP Use Among Trans and/or Non-binary Participants in the TASG Study, a Participatory Study in Germany

**DOI:** 10.1007/s10461-025-04631-z

**Published:** 2025-02-01

**Authors:** Uwe Koppe, Jonas A. Hamm, Chris Spurgat, Alexander Hahne, Robin K. Saalfeld, Manuel Ricardo Garcia, Viviane Bremer, Kathleen Pöge, for the TASG study group, Teo Schlögl, Max Nicolai Appenroth, Né Fink, Heinz-Jürgen Voß, Christoph Schuler, Kuem-Song Plaßmann, Silvia Rentzsch

**Affiliations:** 1 Department of Infectious Disease Epidemiology, Robert Koch Institute, Seestr. 10, 13353 Berlin, Germany; 2 Deutsche Aidshilfe (DAH), Berlin, Germany; 3 Freelance Sex Educator and Bodyworker, Hamburg, Germany; 4 Department of Sociology, Friedrich Schiller University Jena, Jena, Germany; 5 Freelance architect, trans* activist, antiracist activist & photo artist, Munich, Germany; 6 Department of Epidemiology and Health Monitoring, Robert Koch Institute, Berlin, Germany

**Keywords:** Trans, Non-binary, HIV testing, PrEP

## Abstract

HIV testing and pre-exposure prophylaxis (PrEP) are recommended in Germany for individuals at increased HIV risk. However, data on HIV testing, PrEP use, and PrEP knowledge among trans and non-binary people are limited. We analysed data from the ‘Sexuelle Gesundheit in trans und nicht-binären Communitys’ (TASG) study, a participatory study on HIV/STI and sexual health among trans and non-binary people in Germany. The study was designed, promoted, and analysed with active involvement of community members. Participants were invited to complete an anonymous online survey between 1 March and 1 July 2022. The outcomes included HIV testing within the last 5 years, PrEP use, and PrEP-specific knowledge. Predictors for HIV testing were identified using a bootstrap stepwise selection procedure. Among 2468 HIV-negative participants with information on potential HIV risks, 21.5% had potential needs for HIV testing and PrEP. Of these, only 44.3% (208/470, missing: 60) reported testing for HIV within the last 5 years. Older participants, those living in larger cities, and those with higher education levels were more likely to have tested for HIV. Additionally, only 8.3% (38/459, missing: 71) reported ever using PrEP. Among 451 participants with potential PrEP needs (missing: 79), only 57.4% knew at least one of three key PrEP-related facts at the time of the survey. Our findings highlight substantial gaps in HIV testing and prevention among trans and non-binary individuals in Germany with potential needs for these services. Reducing barriers to testing and prevention is essential to enable broader access to these critical services.

**Supplementary Information:**

The online version contains supplementary material available at 10.1007/s10461-025-04631-z.

## Introduction

Transgender/trans and non-binary people are recognised as vulnerable groups for HIV transmission. A systematic review estimated the global standardised HIV prevalence for trans women to be 19.9% (95% confidence interval [CI] 14.7–25.1%) and 2.56% (95% CI 0.0–5.9%) for trans men [1]. These estimates varied across countries and study designs.

Access to and use of HIV testing are essential for early diagnosis and timely initiation of HIV care. However, trans and non-binary people face significant barriers to healthcare and prevention services, including negative healthcare experiences and discrimination [2]. In the 2015 U.S. Transgender Survey, 45% of participants reported never having tested for HIV, with trans women being less likely to test than trans men [3]. In Germany, data from the 2017 European men who have sex with men (MSM) Internet Survey showed that trans MSM were less likely to report HIV testing than cis MSM [4].

HIV pre-exposure prophylaxis (PrEP) is recommended for trans people at increased risk of HIV in both German and U.S. guidelines [5, 6]. However, studies from various countries have shown that a substantial proportion of trans communities are unaware of PrEP [7–9]. In Germany, trans MSM were less likely than cis MSM to have discussed PrEP with a healthcare provider [4]. Importantly, even among those aware of PrEP, the number of participants reporting current PrEP use was low [8, 9]. Enhancing PrEP knowledge and reducing barriers to access are therefore crucial to improving PrEP uptake among trans and non-binary people.

In Germany, data on HIV testing, PrEP knowledge, and PrEP use among trans and non-binary people are limited. To address this, we analysed data from the ‘Sexuelle Gesundheit in trans und nicht-binären Communitys’ (TASG) study, a participatory study on the sexual health of trans and non-binary people in Germany. We investigated the proportion of trans and non-binary participants with a potential need for HIV testing and PrEP based on reported behaviours. Among these participants, we analysed HIV testing within the past 5 years, potential predictors of HIV testing, PrEP use, and PrEP knowledge.

## Methods

### Study Design and Inclusion/Exclusion Criteria

The TASG study is a participatory research project on HIV/STI and sexual health among trans and non-binary persons in Germany, developed and conducted in collaboration with community representatives [10]. The study included an anonymous cross-sectional online survey, created within a participatory framework with the communities. The finalised survey covered topics related to the sexual health of trans and/or non-binary persons and influencing factors (Appendix S1).

Recruitment took place from 1 March to 1 July 2022. Participants were required to consent to participation in the survey, identify as part of the trans and/or non-binary spectrum, be aged ≥ 18 years, and reside in Germany at the time of participation. Because we aimed to investigate HIV testing and PrEP needs, participants with self-reported HIV diagnoses were excluded from this analysis. During social media recruitment, a sharp increase in participation was observed from 28 March to 1 April 2022, with the number of participants rising unexpectedly to several times of what it had been before. This was attributed to trans-hostile responses following a social media post, reflected in some free-text survey comments. Alongside genuine participants, some individuals submitted responses expressing hostile attitudes toward trans and non-binary people. Data cleaning procedures used to remove irrelevant or malicious contributions are described in detail elsewhere [11].

### Outcomes and Variables

One investigated outcome was the potential need for HIV testing and/or PrEP use. This need was assumed for participants who reported one or more of the following behaviours within the last 12 months: ≥ 2 penetrative sex partners, sexualised drug use, and/or offering or using sex work. Penetrative sex using exclusively external devices (e.g., dildos or strap-ons) was not included.

Among individuals with a potential need for HIV testing and/or PrEP use, we examined the outcome of self-reported HIV testing within the last 5 years. Participants who replied ‘no’ to the question ‘Within the last 5 years, have you used an HIV/STI counselling and testing service?’ were classified as not having tested for HIV. Those who answered ‘yes’ were then asked about the frequency of HIV testing. Replies to this question were grouped as ‘at least annually’ and ‘less often/as needed’. The location of the most recent test was analysed under the following categories: community testing centre; public health office; physician; self-test/self-collection test. Reasons for not getting tested for HIV/STI included (multiple answers possible): ‘There are no services near me’, ‘The waiting list for an appointment is too long’, ‘I expected or have already experienced discrimination/stigmatisation’ (e.g., inappropriate treatment, transphobia, racism), ‘I was too scared’, ‘I didn’t know that it’s important for me’, and ‘I haven’t had sex/any risk’. Participants were asked about their condom use and frequency, categorised as always (more than 95% of the time), often (approximately 75% of the time), about half the time (approximately 50%), sometimes (approximately 25% of the time), or never. They were also asked about ever using PrEP, the type of PrEP use (daily, on demand/intermittently, or previous PrEP use), and whether they had prior knowledge of the following statements: ‘In HIV pre-exposure prophylaxis (PrEP), an HIV-negative person takes tablets before and after sex to protect themselves from HIV’, ‘PrEP is approved for use in the form of a daily tablet’, and ‘The effects of hormones are not affected by PrEP’. Participants were considered to have some PrEP knowledge if they indicated that they knew at least one statement before completing the survey.

In our analyses, we included self-reported gender identity categorised as female spectrum, male spectrum, non-binary spectrum, non-binary female spectrum, non-binary male spectrum, and using other terms. Age was analysed in categories of 18–29, 30–39, and ≥ 40 years (the categories 40–49, 50–59, and 60 + years were combined due to sparse data). Potential predictors for obtaining HIV testing included the size of the current residence city (< 100,000; ≥ 100,000 to 1 million; > 1 million inhabitants), education level (low = up to secondary school diploma; medium = high school diploma, apprenticeship; or high = university degree, technician, or master craftsman), and self-reported financial management on monthly income (relatively well, well, and very well grouped as ‘well’; relatively badly, badly, and very badly grouped as ‘badly’). We also included living according to gender identity in daily life (no, yes, or partially); contentment with one’s body (very satisfied and somewhat satisfied coded as ‘yes’; neither satisfied nor dissatisfied, somewhat dissatisfied, and very dissatisfied coded as ‘no’); the number of close social contacts in groups of 0, 1–2 and ≥ 3; and whether one’s identity is respected in daily life (never, sometimes, mostly, or always). Avoidance of medical services because of fear of inappropriate treatment was coded as ‘yes’ if ever reported and ‘no’ if answered ‘never’. Experiencing racism in the healthcare system was coded as ‘yes’ if it had ever been reported and ‘no’ if participants answered ‘never’.

Internalised trans positivity (8 questions) and trans negativity (8 questions) were assessed using the respective summary scores of the Gender Minority Stress and Resilience Measure [12, 13]. Participants scoring between 0–16 were classified as ‘neutral to dismissive’, and those scoring between 17–32 were classified as ‘affirmative’. Depressive symptoms were measured using the PHQ-9 questionnaire [14, 15], which was slightly modified for gender-appropriate language, and anxiety symptoms were assessed with the GAD-7 questionnaire [16, 17]. Participants were categorised as having ‘no to mild’ symptoms (score up to 9), ‘medium’ symptoms (score 10 to 14), and ‘severe’ symptoms (score ≥ 15).

For all variables, answers including ‘Don’t know’, ‘No answer’, or skipping the question were coded as ‘missing’.

### Statistical Analyses

Data were analysed using descriptive methods, with proportions and percentages reported for all categorical variables. Percentages excluded participants with missing values for the variable unless the category ‘missing’ was explicitly included in the percentage calculation. Differences between categorical variables were assessed using Pearson’s chi-square test. Prevalence ratios and 95% CIs were calculated using a Poisson model with robust standard errors to investigate univariable associations. Prevalence ratios were displayed as forest plots. A bootstrap stepwise selection procedure, as described by Sauerbrei and Schumacher, was used to identify and describe predictors for HIV testing [18]. The inclusion frequencies of potential predictors in multivariable logistic regression models were determined using a backward modelling approach with a p-value cut-off of 0.1 after 500 bootstrap replications of the data. Variables included in ≥ 80% of the multivariable models were considered predictors of the outcome.

## Results

**Fig. 1:**
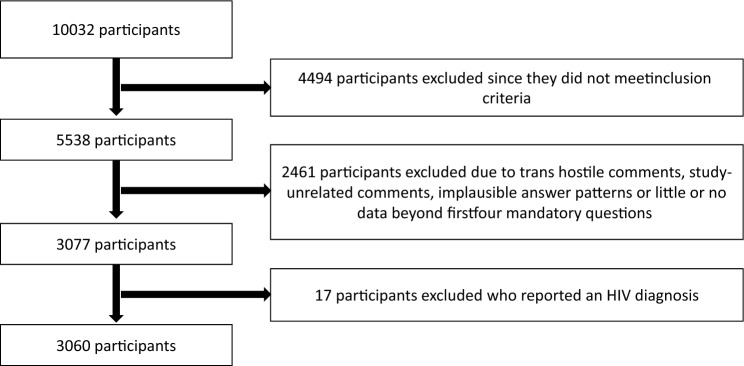
Participant selection for analysis

Overall, 3060 participants who did not report an HIV diagnosis were included in the final dataset (Fig. 1).

We successfully recruited trans and non-binary participants representing different spectra of gender identities (Table 1). More than half of the participants were aged 18–29 years. Regarding sexual behaviour, 44.5% reported no sexual partners with penetration within the last 12 months, while 12.0% reported having two or more partners. Additionally, 7.8% reported sexualised drug use, and 4.4% reported offering or using sex work within the last 12 months.

**Table 1:** Characteristics of participants included in the analysis

	Overall (%)^a^
Total	3060 (100.0)
Gender identity	
Female spectrum	670 (21.9)
Male spectrum	672 (22.0)
Non-binary spectrum	892 (29.2)
Non-binary female spectrum	382 (12.5)
Non-binary male spectrum	391 (12.8)
Using other terms	53 (1.7)
Age category	
18–29 years	1873 (61.2)
30–39 years	766 (25.0)
40 + years	421 (13.8)
Number of sexual partners with penetration last 12 months	
None	1362 (44.5)
1	615 (20.1)
2–3	234 (7.6)
4 +	132 (4.3)
Missing	717 (23.4)
Sexualized drug use	
No	2183 (71.3)
Yes	239 (7.8)
Missing	638 (20.8)
Offered or used sex work within last 12 months	
No	2311 (75.5)
Yes	135 (4.4)
Missing	614 (20.1)

^a^Due to rounding errors, percentages might not add up to 100%

At least one of the questions identifying potential HIV testing and prevention needs was answered by 2468 (80.7%) participants. Based on the number of penetrative sexual partners, sexualised drug use, or offering/using sex work, we identified 530 (21.5%) respondents as having an increased risk of HIV transmission and therefore a potential need for HIV testing and/or PrEP use. Of these, 470 (88.7%) provided information on receiving HIV/STI testing and counselling within the last 5 years.

Of those participants with a potential need for testing and/or PrEP use, only 208 (44.3%) indicated that they had tested for HIV within the last 5 years. The most recent HIV test was most commonly conducted by a treating physician (43.3%, 90/208), followed by community testing centres (29.8%, 62/208), public health offices (16.8%, 35/208), and self-testing or self-collection testing (6.3%, 13/208). Other locations accounted for 2.9% (6/208), and 1.0% (2/208) had missing data. Among participants who tested for HIV within the last 5 years, 101/208 (48.6%) reported testing at least annually, while 107/208 (51.4%) reported testing less frequently or as needed.

**Fig. 2:**
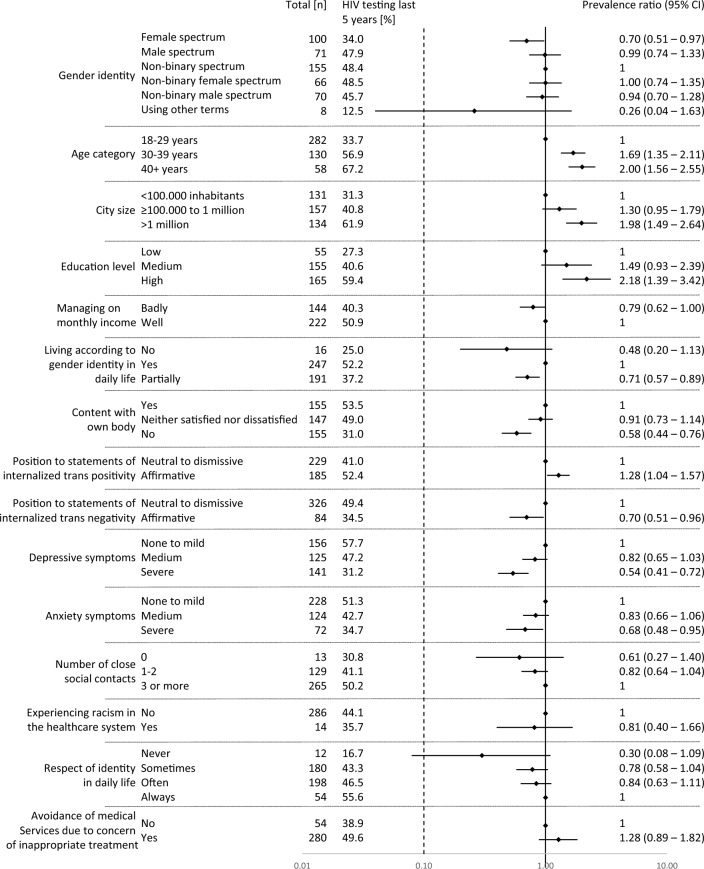
Forest plot of prevalence ratios for HIV testing among people with potential testing needs. 208/470 participants indicated any HIV testing within the last 5 years. Effect estimates are indicated by diamonds and the lines indicate 95% confidence intervals. Effect estimates < 1 indicate a lower prevalence of HIV testing compared to the reference, whereas effect estimates > 1 indicate a higher prevalence. Number of participants with missing data: city size: 48, education level: 95, managing on monthly income: 104, living according to gender identity in daily life: 16, content with own body: 13, internalized trans positivity: 56, internalized trans negativity: 60, depressive symptoms: 48, anxiety symptoms: 46, number of social contacts: 63, experiencing racism in the healthcare system: 170, respect of identity in daily life: 26, avoidance of medical services: 136

Participants aged ≥ 30 years, those living in large cities, and those with higher education levels more frequently reported HIV testing (Fig. 2). A higher proportion of HIV testing was also observed among participants affirming statements of internalised trans positivity. By contrast, participants in the female spectrum had a lower prevalence of HIV testing within the last 5 years than non-binary participants. Participants who reported difficulties managing their current income also reported testing less often. Participants who did not, or only partially, live according to their gender identity in daily life; those who were not content with their own body; and those affirming statements of internalised trans negativity reported HIV testing less frequently. Regarding mental health indicators, participants with severe symptoms of depression or anxiety also reported HIV testing less often. For some estimates, the 95% CI extended beyond 1, indicating that some results might have occurred by chance.

In addition, participants with fewer close social contacts, those who reported experiencing racism in the healthcare system, and those who reported not being respected in daily life indicated numerically less frequent testing for HIV (Fig. 2). However, some strata were small, and the 95% CI extended beyond 1, indicating that these results might have occurred by chance.

Next, we conducted a descriptive analysis to identify potential predictors for obtaining HIV testing. For this, we constructed 500 multivariable models from subsamples of our dataset using bootstrapping. The models were derived using a stepwise backward modelling approach, starting with all variables described in Fig. 2 and retaining those with a p-value of ≤ 0.1. We counted the absolute number of models (out of 500) in which the variables were retained and calculated relative inclusion frequencies by dividing the absolute inclusion frequencies by the total number of bootstrapped models (n = 500). Variables included in ≥ 80% of the models were considered relevant predictors. The results are shown in Table 2. Age and city size were included in 97.4% and 85.8% of the models, respectively, suggesting that they are suitable predictors. The direction of the effect is shown in Fig. 2, indicating that older participants and those living in larger cities were more likely to test for HIV. No other variables were included in ≥ 80% of the models.

**Table 2:** Bootstrap replication inclusion frequencies of potential predictors for obtaining HIV testing within the last 5 years

Covariate	Absolute inclusion frequency	Relative inclusion frequency (%)
Gender identity	260	52.0
Age category	487	97.4
City size	429	85.8
Education level	58	11.6
Managing on monthly income	235	47.0
Living according to gender identity in daily life	317	63.4
Content with own body	76	15.2
Respect of identity in daily life	181	36.2
Avoiding medical services	80	16.0
Experiencing racism in healthcare sector	85	17.0
Depressive symptoms	275	55.0
Anxiety symptoms	314	62.8
Number of close social contacts	327	65.4
Position to statements of internalized trans positivity	116	23.2
Position to statements of internalized trans negativity	129	25.8

Of the 262 participants identified as having a potential HIV risk but who did not obtain HIV/STI testing and counselling within the last 5 years, 255 (97.3%) provided one or more potential reasons. The most common reason was being ‘too scared’ (46.3%), followed by considering themselves not at risk (29.8%) and not considering HIV/STI counselling as important (27.1%). Discrimination and stigmatisation also played a relevant role: 28.6% of participants reported having experienced or expecting discrimination/stigmatisation. Among these, 67.1% (49/73) reported avoiding medical services because of fear of inappropriate treatment, and 5.5% (4/73) reported experiencing racism in the healthcare system. Access barriers were also relevant, including the unavailability of testing in their area (16.1%) and excessively long waiting times for an appointment (5.9%).

We analysed safer sex practices among the 530 respondents identified as having a history of sexual encounters with a risk for HIV transmission. Condoms were used by 335 (74.8%, missing: 82) respondents; however, only 191 (57.0%) reported always using them. Fewer participants reported using condoms often (in approximately 75% of cases, 22.1%), about half the time (9.3%), or sometimes (in approximately 25% of cases, 9.6%; missing: 2.1%). PrEP had been used by only 38 (8.3%, missing: 71) respondents, of whom 15 (39.5%) used it daily, 13 (34.2%) used it on demand or intermittently, and 8 (21.1%) had used PrEP in the past (missing: 2). PrEP use was numerically lower among participants who reported avoiding medical services because of concerns about inappropriate or discriminatory treatment compared to those who did not (6.7% [19/283] vs. 14.0% [8/57], Pearson chi-square = 3.48, p = 0.062). Of the 335 participants providing data on both condom and PrEP use, 130 (38.8%) reported not always using condoms and no PrEP use.

Among participants with potential PrEP needs, 457 answered questions on PrEP knowledge. While 47.9% were aware that PrEP is a pill taken by HIV-negative persons before and after sex for protection against HIV, 45.9% (missing: 4) knew that PrEP is approved for use as a daily tablet, and 24.5% (missing: 4) were aware that the effects of gender-affirming hormone therapy are not impacted by PrEP. Only 20.2% of participants knew all three facts, but 57.4% were knowledgeable about at least one statement at the time of the survey (missing: 6).

**Fig. 3:**
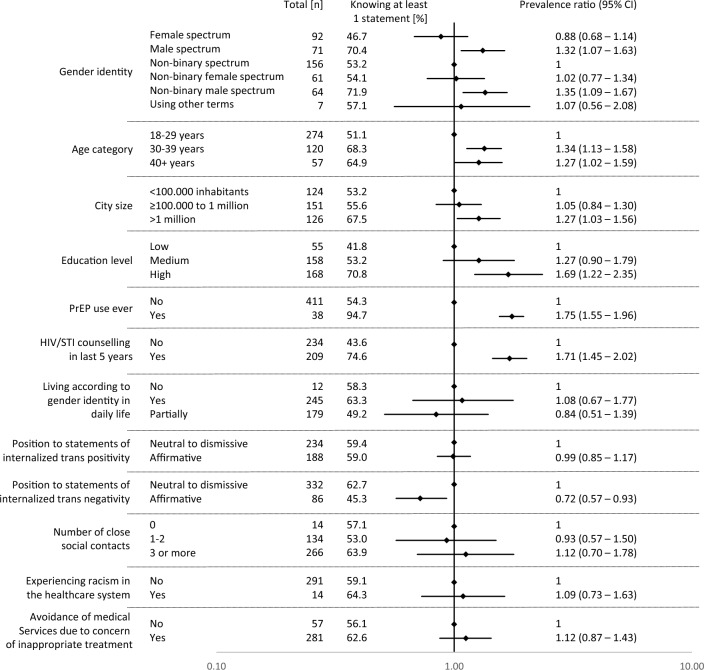
Forest plot of prevalence ratios for PrEP knowledge among people with potential PrEP needs. Effect estimates are indicated by diamonds and the lines indicate 95% confidence intervals. Effect estimates < 1 indicate a lower prevalence of knowing at least one PrEP fact compared to the reference, whereas effect estimates > 1 indicate a higher prevalence. Number of participants with missing data: city size: 50, education level: 70, ever used PrEP: 2, HIV/STI counselling last 5 years: 8, living according to gender identity: 15, internalized trans positivity: 29, internalized trans negativity: 33, number of social contacts: 37, experiencing racism in the healthcare system: 146, avoidance of medical services: 113

Knowledge about PrEP was more prevalent among participants in the male and non-binary male spectrum, as well as among older participants (Fig. 3). Additionally, those who had ever used PrEP, received HIV/STI testing and counselling within the last 5 years, lived in a large city, or had a high education level more frequently reported knowing at least one statement about PrEP (Fig. 3). By contrast, participants who affirmed statements of internalised trans negativity were less likely to know at least one statement about PrEP (prevalence ratio = 0.72; 95% CI 0.57–0.93). We found no significant difference in PrEP knowledge among participants who reported experiencing racism in the healthcare system (Fig. 3). Furthermore, participants who avoided medical services due to concerns about inappropriate or discriminatory treatment had numerically more PrEP knowledge, although the confidence interval extended beyond the null value, suggesting this might have been a chance finding.

## Discussion

Within the participatory TASG study, we successfully recruited a large number of trans and non-binary participants in Germany and conducted quantitative analyses on critical public health issues such as HIV testing, PrEP uptake, and PrEP knowledge. The study was designed and conducted in collaboration with representatives from the trans and non-binary communities, ensuring that the question design and language were appropriate and that there was community support for the study.

Among participants with a history of potential HIV transmission risks, only 44.3% had been tested within the last 5 years, and only 8.3% reported ever using PrEP. We identified significant gaps in knowledge about PrEP, with only 57.4% of respondents aware of at least one of three key PrEP-related statements prior to the survey and just 20.2% aware of all three facts.

Our finding that 44.3% of participants reported HIV testing within the last 5 years aligns with previous studies. For comparison, a German study showed that 65.1% of 63 trans participants had never tested for HIV [19]. Similar proportions of participants not testing for HIV were reported in the 2015 U.S. Transgender Survey, a Canadian study in Ontario, and an online survey in the United Kingdom [3, 20, 21]. In our study, trans women were the least likely to report HIV testing compared with other gender identity categories, echoing findings from the 2015 U.S. Transgender Survey, where trans women were more likely to be never tested compared to trans men [3]. Conversely, another U.S. study found that trans masculine and non-binary individuals assigned female at birth more frequently reported never testing for HIV [22], while the Transgender Stress and Health Study noted that non-binary participants were less likely to report HIV testing within the last year than were trans male and trans female participants [23]. These discrepancies highlight how access to HIV testing varies across contexts and subgroups within trans and non-binary communities. In Germany, few testing opportunities are specifically tailored to trans and non-binary people [24]. Male-identified trans and non-binary individuals may be more likely to access testing services targeted at MSM, potentially explaining their higher proportions of HIV testing. This underscores the need for information campaigns tailored to trans and non-binary individuals, providing targeted information for people of all gender identities.

Access to HIV testing relies on the availability of trans-inclusive testing facilities within reachable distances. In our study, HIV testing was highest among participants living in large cities, where trans- and non-binary-inclusive HIV/STI testing services are more commonly available [24]. However, negative experiences can discourage individuals from accessing these services. In the qualitative part of the TASG study, some participants reported that while certain anonymous testing sites claimed to be inclusive, they lacked basic knowledge about trans and non-binary individuals [10]. Expanding inclusive and knowledgeable HIV/STI counselling sites to more areas could help reduce these accessibility barriers. Similarly, a qualitative study on barriers to HIV and STI testing suggested that access to trusted providers facilitates testing among trans MSM and proposed integrating HIV/STI counselling and testing with routine monitoring of gender-affirming hormone therapy to increase uptake [25]. The qualitative part of the TASG study also highlighted self-collection testing as a viable option to provide accessible testing opportunities without exposing individuals to stigmatising environments [10]. Additionally, a randomized controlled trial conducted in England and Wales demonstrated that offering free HIV self-testing increased testing uptake among trans men and trans women [26]. However, the requirement for co-payment in self-testing or self-collection services may deter individuals with lower incomes. In our study, participants experiencing financial difficulties were less likely to test for HIV, emphasising the importance of designing testing strategies that account for the financial constraints faced by trans and non-binary communities.

Age was identified as a predictor for HIV testing in our analyses, with older participants more frequently reporting testing. This finding aligns with results from other studies [3, 21]. In our context, it is plausible that younger individuals may be at earlier stages of transition, have less disposable income, or face competing priorities in their lives, all of which could deter them from undergoing HIV testing. Additionally, older participants may have had more opportunities for testing over time and might still be influenced by experiences from the early phase of the HIV epidemic, making them more likely to engage in testing. Future studies should investigate the specific vulnerabilities and barriers faced by young trans and non-binary individuals in greater detail.

Individuals with potential HIV testing needs also require awareness that testing might be beneficial to them. Nearly one-third of participants in our study did not access HIV/STI testing and counselling because they perceived their risk as not high enough, despite reporting behaviours indicative of a potential need for testing. Similarly, low perceived risk was the most common reason for not testing in the 2015 U.S. Transgender Survey [3]. This underscores the importance of targeted communication to trans and non-binary communities, emphasising the benefits of HIV testing and the accurate assessment of HIV transmission risks.

Participants who lived according to their gender identity in daily life and were content with their bodies reported HIV testing more frequently. In the qualitative part of the TASG study, transitioning and coming out were identified as potential empowering factors that enabled participants to explore their sexuality [10]. Additionally, internalised trans positivity–a positive attitude toward one’s own gender identity–was associated with a higher prevalence of HIV testing. These findings suggest that factors empowering trans and non-binary individuals in their daily lives may positively influence HIV testing uptake.

The social environment also plays a crucial role as a source of information and support. In our study, participants with three or more close social contacts reported the highest proportion of HIV testing compared to those with fewer close social contacts. A recent review suggested that leveraging social networks for peer support and encouragement can be an effective strategy to promote HIV testing [27]. Therefore, expanding professional peer-to-peer education on HIV testing could increase knowledge within the communities.

Participants with medium to severe symptoms of anxiety or depression, as well as those experiencing internalised trans negativity, were less likely to report HIV testing. This highlights the importance of mental health support and gender-affirming services in increasing testing uptake. Experiences of discrimination can also negatively affect access to healthcare services [2]. While the associations between avoidance of healthcare services or experiencing racism in the healthcare system and HIV testing in our analyses are were inconclusive due to the confidence intervals extending beyond the null value, international literature consistently identifies stigma, discrimination, and racism as significant barriers to HIV testing [2, 8, 28, 29]. Thus, testing services should aim to reduce these barriers.

Among participants with potential PrEP needs, only 8.3% reported ever using PrEP, and just 57.4% were aware of at least one of three key PrEP-related statements prior to the survey. The low PrEP use among these participants is particularly concerning for those who do not always use condoms. While access to and use of PrEP varies by context and has generally been low in studies from various countries [7–9, 19, 30–33], targeted programs for trans communities have demonstrated higher PrEP uptake [34, 35]. We found weak evidence suggesting that participants avoiding medical services due to concerns about inappropriate or discriminatory treatment reported lower PrEP use; however, this finding may have occurred by chance due to small numbers. Additionally, experiences of discrimination and racism in the healthcare system, along with inadequate provider training in areas such as trans awareness, antiracism, and disability accessibility, have been identified as barriers to PrEP use in other studies [36, 37]. Establishing a network of trans-inclusive, non-discriminatory, and accessible providers could help improve PrEP uptake. Integrating PrEP provision with gender-affirming care such as monitoring gender-affirming hormone therapy, has also been proposed as a strategy to increase uptake [38–40]. However, access to trans-inclusive PrEP prescribers often depends on the area of residence, highlighting the need for more inclusive strategies, such as telemedicine, to improve access.

Another important barrier to PrEP uptake is lack of knowledge. A study from a trans-inclusive testing site in Berlin, Germany, identified insufficient information about PrEP as a common reason for its non-use [24]. In our study, 47.6% of participants with potential PrEP needs were unaware of the three PrEP-related statements before participating. This aligns with findings from a previous study in Germany, where trans MSM demonstrated lower PrEP knowledge than cis MSM [4]. Similarly, other studies among trans individuals have reported substantial proportions of participants unaware of PrEP [8, 9, 41]. In our study, PrEP knowledge was more common among older participants, those who had received HIV/STI testing and counselling within the last 5 years, those living in larger cities, and those with higher education levels. To improve PrEP knowledge, information campaigns targeting trans and non-binary people across all gender identities, educational backgrounds, and regions are needed. Social networks and social media, recognised as key information sources for trans and non-binary communities, could play a pivotal role in delivering this information [7, 42]. Professional peer-navigators can also enhance outreach by disseminating information and supporting their peers in accessing PrEP [43, 44].

In this study, we successfully recruited a large and diverse sample of trans and non-binary individuals in Germany and conducted quantitative analyses on critical public health issues, including HIV testing, PrEP uptake, and PrEP knowledge. The study was developed and implemented in collaboration with representatives from the trans and non-binary communities, ensuring that the question design and language were appropriate and that the study had strong community support.

Some limitations should be considered when interpreting the results of our analyses. Due to the nature of the study, we relied on a convenience sample of trans and non-binary individuals, which may be subject to selection bias. For example, more than half of the participants were under 30 years old, potentially limiting the generalisability of our findings to older trans and non-binary populations. Additionally, during recruitment, we encountered a wave of trans-hostile comments and calls for fake participation aimed at manipulating and invalidating the study results, necessitating extensive data cleaning (see Fig. 1). While it is possible that some hostile participants remained in the final dataset or that some genuine participants were inadvertently removed, the consistency of our findings with previously published studies supports the assumption that our data cleaning process did not introduce significant distortions. Another limitation is that we did not collect information about the prevention strategies of participants’ sexual partners, meaning that some participants may have been classified as having a potential HIV risk despite using appropriate prevention measures (e.g., consistent and successful condom use, partners on PrEP, or treatment as prevention). As a result, the number of participants identified as having HIV risks may be an overestimate.

## Conclusion

We identified substantial gaps in HIV testing, PrEP use, and PrEP knowledge among trans and non-binary individuals in Germany with a potential need for these services. Addressing these gaps requires reducing barriers to access by ensuring the availability of trans-inclusive, antiracist, and disability-accessible services, disseminating targeted testing and prevention information to trans and non-binary communities, and strengthening community structures that support peer education.

## Supplementary Information

Below is the link to the electronic supplementary material.

Supplementary file1 (DOCX 56 KB)

## Data Availability

Data from this study are available from the corresponding author upon reasonable request.
