# Supplementary material for: HIV Testing and PrEP Use Among Trans and/or Non-binary Participants in the TASG Study, a Participatory Study in Germany
Source: AIDS Behav. 2025 Feb 1;29(5):1608–18. doi: 10.1007/s10461-025-04631-z (PMC12031786; doi:10.1007/s10461-025-04631-z)
Supplement: Supplementary file 1 — Supplementary file1 (DOCX 56 KB) [file 10461_2025_4631_MOESM1_ESM.docx]

**HIV testing and PrEP use among trans and/or non-binary participants in the TASG study, a participatory study in Germany – Appendix**

In the following, we report the questions of the survey, which were analysed in this manuscript. The whole questionnaire can be found in the appendix of the following publication:

Mario Martín-Sánchez, Kathleen Pöge, Alexander Hahne, Jonas Hamm, Viviane Bremer, Uwe Koppe & The TASG-study group. Discrimination based on gender identity and decision-making regarding HIV/STI-protected sex, a cross-sectional study among trans and non-binary people in Germany. BMC Public Health, Volume 24, Article number: 3013 (2024)

| In order to enable statistical evaluation, we have to group a range of different terms into a handful of categories. In the following, we have listed some categories in which various terms are combined to form spectrum. Not all terms within a spectrum need to be relevant to you, or you may reject certain terms in relation to yourself.  On which of the following spectrums would you be most likely to locate yourself? | **Female spectrum** (woman, transfeminine, transgender* woman, MtF, transsexual woman etc.  **Male spectrum** (male, transmasculine, transgender* male, FtM, transsexual male etc.)  **Non-binary, female spectrum** (both non-binary and female spectrum)  **Non-binary, male spectrum** (both non-binary and male spectrum)  **Non-binary spectrum** (abinary, polygender, genderfluid, gender non-conforming, both male and female, genderqueer etc.)  I use other terms, but I can basically assign the generic term transgender* and/or non-binary to myself  I do not locate myself on the transgender* or non-binary spectrum |
| --- | --- |
| How old are you? | Younger than 18  18-29  30-39  40-49  50-59  60 or older |
| Which description applies to where you currently live? | Metropolis (over 1 million inhabitants)  City (up to 1 million inhabitants)  Town (up to 100,000 inhabitants)  Small town (up to 20,000 inhabitants)  Countryside/village (up to 5,000 inhabitants)  I live in changing accommodation in different places  No answer  Don't know |
| Do you live in accordance with your gender identity in your everyday life? | Yes  Partly (e.g. only on certain days, at certain times or in certain places)  No  No answer  Don't know |
| People can perceive their gender identity as corresponding more or less with their body.  In your own personal perception: How satisfied are you with your body in terms of your gender identity? | Very satisfied  Somewhat satisfied  Neither satisfied nor dissatisfied  Somewhat dissatisfied  Very dissatisfied  No answer  Don’t know |
| In general, how often is your gender identity respected? | Never  Sometimes  Mostly  Always  No answer  Don’t know |
| People can live their sexuality in different ways. In the following section, we are interested in sexual encounters which involve mutual contact of mucous membranes. This includes rubbing mucous membranes together as well as oral sex (e.g. licking, sucking) and penetration (e.g. fucking).  It doesn’t matter whether barriers (e.g. condoms, dental dams) are used.  For this question, we are not counting French kissing (e.g. kissing with tongues) as sexual contact.    Within the last 12 months, have you had any sexual encounters that match the description above? | Yes  No  Don't know |
| Within the last 12 months, with how many different people have you had sexual encounters that resulted in mucous membrane contact? | 0  1  2-3  4-5  6-10  11-20  More than 20  Don't know |
| Sex that involves penetration (e.g. fucking) can play a particular role in the transmission of certain STIs, such as HIV.  This does not include penetrative sex with external aids such as strap-ons or externally attached genitalia.  In the past 12 months, have you had sex that resulted in penetration? | Yes  No  Don't know |

| Within the last 12 months, with how many different people have you had penetrative sex (e.g. fucking)? | 0  1  2-3  4-5  6-10  11-20  More than 20  Don't know |
| --- | --- |
| Within the last 12 months, have you had sex while under the influence of drugs?  (e.g. ecstasy/MDMA, cocaine, amphetamines (speed), methamphetamine (crystal, meth, tina, pervitin), mephedrone or ketamine) | Yes  No |
| Some people make use of sex workers or engage in sex work themselves. Since this can be a part of people’s lived sexuality, we have included it in this survey.    Within the last 12 months, have you paid for sex?  By payment we mean the exchange of money, gifts or favours (housing, groceries, etc.) for sex. | Yes  No  No answer |
| Within the last 12 months, have you been paid for sex?    By payment we mean the exchange of money, gifts or favours (housing, groceries, etc.) for sex. | Yes  No  No answer |
| Within the last 5 years, have you used an HIV/STI counselling and testing service? | Yes  No  No answer |
| Why haven’t you used an HIV/STI counselling and testing service?  Please select all the answers that apply: | There are no services near me  The waiting list for an appointment is too long  I expected or have already experienced discrimination/stigmatisation  I was too scared  I didn't know that it’s important for me  I haven’t had sex/no risk  Don't know |
| What was the last HIV/STI counselling and testing service that you used? | Checkpoint/Aidshilfe  Local health authority  Family doctor/GP  Specialist medical practice/HIV practice  HIV self-tests  Send-in tests  Other HIV/STI counselling and testing service, namely: [free text] |

| You have stated that you have made use of HIV/STI counselling or testing services within the last 5 years.  Within the last 5 years, how often have you been tested for HIV and/or other sexually transmitted infections such as syphilis?    HIV  Syphilis (lues)  Gonorrhea (tripper, the clap)  Chlamydia  Hepatitis C  Human papillomavirus (HPV test/pap smear) | At least once every 6 months  At least once a year  At least once every 2 years  At least once every 5 years   Never  When I need to  Don't know |
| --- | --- |
| Have you ever been diagnosed as HIV positive? | Yes  No  Don't know  No answer |
| What precautions against HIV and other sexually transmitted infections (STIs) are you currently using to protect yourself?    Please select all answers that apply to you | I use condoms (internal or external condoms).  I use dental dams.  I use gloves.  I have sex with people who have proved that they don’t have STIs or HIV.  I use PrEP (pre-exposure prophylaxis).  I have sex with people who are HIV negative and who are taking PrEP.  I have sex with people who are HIV positive and who are below the viral load detection limit.  I avoid situations with a high risk of infection (e.g. sex under the influence of drugs).  I get tested regularly.  I use PEP (post-exposure prophylaxis).  I avoid sexual contact.  I avoid certain sexual practices (e.g. penetration).  I don’t (or very rarely) change sexual partners.  Other:_____ |
| How often do you use condoms during genital penetrative sex?    By genital penetrative sex we mean sexual practices in which the genital mucous membranes are involved in penetration (e.g. fucking). This also includes anal intercourse. It does not include, for example, genital penetration with strap-ons/externally attached genitals or oral sex. | Always  Often (approx. 75% of the time)  About half the time (approx. 50%)  Sometimes (approx. 25% of the time)  Never  Don't know  No answer |

| Did you know?    The following three statements are APPLICABLE.    In HIV pre-exposure prophylaxis (PrEP), an HIV-negative person takes tablets before and after sex to protect themselves from HIV.    PrEP is approved for use in the form of a daily tablet.  The effects of hormones are not affected by PrEP. | I knew that  I wasn’t sure about that  I didn't know that  I don’t understand that  I don’t believe that’s true  No answer |
| --- | --- |
| Have you ever received PrEP? | Yes  No  Don't know  No answer |
| Are you currently taking, or have you ever taken, PrEP? | Yes, I’m currently taking PrEP every day  Yes, I take PrEP at certain times when I need it (on demand or daily during certain periods.  Yes, I have taken PrEP once, but I am no longer taking it  No  Don't know  No answer |
| How often have you felt affected by the following complaints during the last 2 weeks?    Little interest or enjoyment in your activities  Depression, melancholy or hopelessness  Difficulty falling asleep, sleeping through the night, or sleeping too much  Fatigue, or the feeling of having no energy  Lack of appetite or overwhelming desire to eat  A poor opinion of yourself; the feeling of being a failure or a disappointment to your family  Difficulty concentrating, for example when reading the newspaper or watching television  Have your movements or speech slowed down so much that it is noticeable to others? Or, on the contrary, have you been “fidgety” or restless, and thus with a stronger urge to move than usual?  Thoughts that you would rather be dead or feelings of self-harm  *Reference: Spitzer, R. L. Kroenke, K. Williams, J. B. (1999). Validation and utility of a self-*  *report version of PRIME-MD: The PHQ primary care study. JAMA, 282, 1737– 1744* | Not at all  On some days  On more than half the days  Nearly every day |

| How often have you felt affected by the following complaints during the last 2 weeks?  Nervousness, anxiety or tension  Not being able to stop or control your worrying  Worrying excessively about various matters  Difficulty relaxing  Restlessness, making it difficult to sit still  Feeling irritable or quick to lose your temper  Feeling afraid, as if something bad is about to happen  *Reference: Spitzer, R. L., Kroenke, K., Williams, Williams, J. B. W., & Löwe, B. (2006). A brief measure*  *for assessing generalized anxiety disorder. Archives of Internal Medicine, 166, 1092–1097.* | Not at all  On some days  On more than half the days  Nearly every day |
| --- | --- |
| The following section is about social relationships and contacts.  How many people are close enough friends for you to rely on them when you have serious personal problems? | None  1 to 2  3 to 5  6 or more  Don't know  No answer |
| In the next question, we would like to learn more about your attitudes towards your gender identity.  Please indicate the extent to which you agree with the following statements:  My gender identity makes me feel special and unique.  I don’t mind telling people that my gender identity is different from the sex I was assigned at birth.  I’m happy to talk about my gender identity with practically anyone.  For me it’s a gift that my gender identity is different from the sex I was assigned at birth.  I'm like other people, but I'm also special because my gender identity is different from the sex I was assigned at birth.  I’m proud to be someone whose gender identity is different from the sex they were assigned at birth.  I have no problem revealing to other people that my gender identity is different to the sex I was assigned at birth.  I would prefer that people knew everything and accepted me, including my gender identity and gender history.  *Reference: Testa, R. J., Habarth, J., Peta, J.,*  *Balsam, K., & Bockting, W. (2015). Development of the Gender Minority Stress and Resilience Measure. Psychology of Sexual Orientation and Gender Diversity, 2(1), 65–77. https://doi.org/10.1037/sgd00000*  *81* | Completely disagree  Somewhat disagree  Neutral  Agree  Completely agree |

| Please indicate the extent to which you agree with the following statements:  My gender identity bothers me.  My gender identity makes me feel like a freak.  When I think about my gender identity, I feel depressed (decreased motivation, loss of interest, joylessness).  When I think about my gender identity, I feel unhappy.  Because of my gender identity, I feel like an outcast.  I often ask myself: Why can't my gender identity just be "normal"?  I find my gender identity embarrassing.  I envy people who don't have the same gender identity as me.  *Reference: Testa, R. J., Habarth, J., Peta, J.,*  *Balsam, K., & Bockting, W. (2015). Development of the Gender Minority Stress and Resilience Measure. Psychology of Sexual Orientation and Gender Diversity, 2(1), 65–77. https://doi.org/10.1037/sgd00000*  *81* | Completely disagree  Somewhat disagree  Neutral  Agree  Completely agree |
| --- | --- |
| When was the last time you had the following experiences in the medical sector?  I didn’t take advantage of certain medical services because I was concerned that I would be treated inappropriately.  My concerns were not taken seriously.  I was under the impression that practitioners’ personal views and attitude influenced diagnoses or treatment decisions.  I had to inform a practitioner about transgender or non-binary issues (e.g. medical or legal aspects, life situation).  I was asked inappropriate questions in a medical consultation or during medical treatment.  I was addressed by the wrong name or the wrong pronoun.  I was refused a diagnosis.  I received a stigmatising misdiagnosis.  I was given a diagnosis. It took me a lot of time and energy to understand that it was a misdiagnosis and to distance myself from it.  I was under the impression that hasty conclusions were drawn and premature associations were made, needs or fears were implied.  I was denied or offered delayed access to medical or therapeutic services, or I was not offered them at all.  I experienced racism (disadvantages when making appointments, rudeness and disrespect, health consequences caused by racism/the stress of belonging to a minority group being ignored, rough treatment, refusal of treatment due to assumed characteristics (e.g. reduced sensitivity to pain), indoctrination, refusal to issue sick notes, pain not taken seriously, misdiagnoses due to Eurocentric training (e.g. failure to recognise skin diseases in people with a different skin colour), having to justify why I had not had medical treatment in my “country of origin".).  I was told I was too fat to have an operation.  My doctors were not specialised in treating transgender and non-binary people.  I had to change doctors because I didn’t feel they were competent to deal with my issues, but they didn't want to do any training.  I was mistreated.  I was threatened that word of my case would get around among practitioners.  Other medical diagnoses were linked to my gender identity.  My identity as a transgender* or non-binary person was not taken seriously because I have a psychiatric diagnosis.  My identity as a transgender* or non-binary person was not taken seriously because I am “too young”.  My identity as a transgender* or non-binary person was not taken seriously due to racism.  My identity as a transgender* or non-binary person was not taken seriously due to my religion/worldview.  My identity as a transgender* or non-binary person was not taken seriously because I have a disability or am disabled by society.  I was told that I had to get a grip on other “health problems” (e.g. depression, obesity) before I could transition.  Other, namely: ________  (You can enter further experiences in the free text field) | Never  Within the last 4 weeks  Within the last 6 months  Within the last 12 months  Within the last 5 years  More than 5 years ago  Don't know  No answer |
| In the last section of the survey, we will ask you 8 questions about your life circumstances. Depending on your answers, you may be asked up to 2 follow-up questions. If you don’t want to answer certain questions, simply click on “Next”.  What is your highest school-leaving qualification?  If you didn’t go to school in Germany, please give the answer that most closely corresponds to your school-leaving certificate. | I left school without a certificate  Volks-/Hauptschulabschluss (elementary/lower secondary school leaving certificate/GDR year 8)  Realschulabschluss/Mittlere Reife (intermediate/general secondary school leaving certificate/GDR year 10)  Fachhochschulabschluss (technical college certificate)  Abitur (general qualification for university entrance)  Other school leaving qualification: [free text]  I am currently at school/in vocational training  Don't know  No answer |
| Which vocational training or technical/university degrees do you have?    If you didn’t receive vocational training or go to technical college/university in Germany, please give the answer that most closely corresponds to your qualifications. | In-house vocational training (apprenticeship)  Master craftsman, technician or equivalent technical college qualification  Bachelor’s degree  Master's degree, diploma, engineering degree, German state or teaching examination (e.g. studies to be a doctor, teacher, pharmacist, food chemist, psychotherapist, jurist or surveyor)  PhD/doctorate  Other professional qualification: _________  I am currently a trainee/apprentice  I am currently a student  I have no professional qualifications  Don't know  No answer |
| How do you manage on your monthly income? | Very badly  Badly  Relatively badly  Relatively well  Well  Very well |
